# Social Inequities in Urban Heat and Greenspace: Analyzing Climate Justice in Delhi, India

**DOI:** 10.3390/ijerph18094800

**Published:** 2021-04-30

**Authors:** Bruce C. Mitchell, Jayajit Chakraborty, Pratyusha Basu

**Affiliations:** 1 National Community Reinvestment Coalition (NCRC), Washington, DC 20005, USA; bmitchell@ncrc.org; 2 Department of Sociology & Anthropology, University of Texas at El Paso, El Paso, TX 79968, USA; pbasu@utep.edu

**Keywords:** environmental justice, climate justice, urban studies, urban heat island, greenspace, remote sensing

## Abstract

Climate change and rapid urbanization currently pose major challenges for equitable development in megacities of the Global South, such as Delhi, India. This study considers how urban social inequities are distributed in terms of burdens and benefits by quantifying exposure through an urban heat risk index (UHRI), and proximity to greenspace through the normalized difference vegetation index (NDVI), at the ward level in Delhi. Landsat derived remote sensing imagery for May and September 2011 is used in a sensitivity analysis of varying seasonal exposure. Multivariable models based on generalized estimating equations (GEEs) reveal significant statistical associations (*p* < *0*.05) between UHRI/NDVI and several indicators of social vulnerability. For example, the proportions of children (β = 0.922, *p* = 0.024) and agricultural workers (β = 0.394, *p* = 0.016) are positively associated with the May UHRI, while the proportions of households with assets (β = −1.978, *p* = 0.017) and households with electricity (β = −0.605, *p* = 0.010) are negatively associated with the May UHRI. In contrast, the proportions of children (β = 0.001, *p* = 0.633) and agricultural workers (β = 0.002, *p* = 0.356) are not significantly associated with the May NDVI, while the proportions of households with assets (β = 0.013, *p* = 0.010) and those with electricity (β = 0.008, *p* = 0.006) are positively associated with the May NDVI. Our findings emphasize the need for future research and policies to consider how socially vulnerable groups are inequitably exposed to the impact of climate change-related urban heat without the mitigating effects of greenspace.

## 1. Introduction

Global climate change is causing an increase in the temperature baseline in economically developing regions that contain some of the densest and most rapidly urbanizing cities so that ‘a substantial portion of humanity will be exposed to mean annual temperatures warmer than nearly anywhere today’ [1]. This trend has raised concerns about climate injustice—the extent to which socially vulnerable groups are more likely to be exposed to the negative consequences of elevated temperatures while also being less likely to mitigate its harmful effects [2,3]. Climate justice activists and scholars have thus emphasized the need to ‘recognise humanity’s responsibility for the impacts of greenhouse gas emissions on the poorest and most vulnerable people in society by critically addressing inequality and promoting transformative approaches to address the root causes of climate change’ [4] (p. 3). In large metropolitan regions, the formation of urban heat islands is one major cause of rising temperatures [5,6,7]. These occur due to the structural density of land cover in cities where buildings and impervious surfaces, such as concrete and asphalt, retain and re-radiate thermal energy while preventing evapotranspiration, causing a localized intensification of atmospheric heat [8]. While greenspaces are one way to counter urban heat islands, previous studies have shown that socially vulnerable populations face greater urban heat exposure [9] as well as reside in areas that lack vegetation [10].

India is an especially useful context for examining climate injustice since a significant increase in mean temperature between 1986 and 2015 has been recorded [11] (p. 22). Furthermore, several climate models project that heatwaves in India will occur earlier in the year, last longer, and increase in both frequency and intensity [12,13,14,15,16]. While prolonged exposure to temperatures above 35 °C has the potential to exceed human adaptive capacity, the highest temperatures during heatwaves in India are likely to go well beyond this and reach around 50 °C [16]. As population and built-up surface continue to expand in major urban areas in India [17,18], it becomes imperative to analyze whether socially vulnerable populations reside in areas exposed to higher levels of heat.

Previous studies on social vulnerability to heat in urban India provide variables pertinent to understanding climate injustice. These studies have identified neonatal infants, pregnant women, children under 15 years and people over 65 years as socio-demographic groups that are especially vulnerable to heat [19,20]. Some occupations, such as outdoor laborers and construction workers, are exposed to the full impact of extreme heat events due to the physical exertion of their work [21,22]. Informal settlements or slums concentrate social vulnerabilities due to overcrowding, poor quality housing, inadequate sanitation, and lack of access to drinkable water [23,24,25,26]. Moving beyond single indicators, two studies are noteworthy in their conceptualization of heat vulnerability in composite terms through indicators that represent (a) exposure, such as degree of heat, and poverty; (b) sensitivity/susceptibility, such as social and health characteristics, housing condition, and unemployment; and (c) adaptive capacity, such as electricity, literacy, and television ownership [27,28]. Additionally, recent environmental justice studies in India have utilized Census data to measure social disadvantage through caste and tribal status, literacy, and housing assets and amenities [29,30,31,32].

Delhi, India’s capital and second largest city by population, has been widely studied in terms of the spatial distribution of heat [33,34,35,36,37,38]. The severity of the situation here is exemplified by a heatwave in May 2015 during which temperatures rose beyond 44 °C, which reportedly was hot enough to melt pavements [13]. Studies have linked the spatial distribution of heat in Delhi to social vulnerability through a focus on specific neighborhoods. One study on exposure to outdoor heat in three major South Asian cities, including Delhi, compared high and low-income neighborhoods along a selected transect in each city [39]. Collating data from mobile measurements and stationary sources, it was found that low to middle income neighborhoods were more likely to show compact settlement patterns and hence higher heat stress. Another study in East Delhi drew attention to how the characteristics of neighborhoods in terms of their economic activities and social composition shape exposure to heat [40]. This focus on selected areas can be extended through analyses which encompass the entire city, and build a more comprehensive portrayal of heat vulnerability at the urban scale.

In terms of greenspace, the presence of forested and riverine areas in the central and eastern parts of Delhi, as well as agricultural areas in the northern and western peripheries, can be considered to provide protection from heat to those residing in close proximity [41]. While a study in the city of Mumbai found neighborhoods of higher socioeconomic status to be more proximate to greenspace [42], similar studies have yet to be conducted in other cities in India.

This article focuses on Delhi to understand the climate justice implications of urban heat and greenspace distribution. Specifically, we seek to determine if socially vulnerable groups in Delhi are disproportionately exposed to urban heat, while also residing in areas with relatively little greenspace. The main research questions for this study are: (1) How does the spatial distribution of urban heat and greenspace in May (pre-monsoon) relate to the spatial distribution of socially vulnerable groups in Delhi’s neighborhoods? (2) How does this relationship change with seasonal variations in temperature, as denoted by the distribution of urban heat and greenspace in September (post-monsoon)? For the first question, we hypothesize that socially vulnerable groups will be more likely to reside in neighborhoods with higher urban heat and lower vegetation in May, so that a situation of climate injustice will become visible in Delhi. The second question then seeks to examine the extent to which this greater exposure to urban heat and lack of vegetation continues in September, or is mitigated due to monsoonal conditions.

Our study of Delhi adds to analyses of climate justice in India in three main ways. First, by focusing on urban heat and greenspace, we consider how social vulnerability in urban landscapes is shaped in terms of both burdens and benefits. This expands existing studies by being attentive to the difference between relatively more and less urbanized areas, an aspect which becomes important in the case of Delhi which is located within India’s northern agricultural belt. Second, our study of Delhi complements studies which focus on the state level in India [27], or selected neighborhoods within the city [39,40]. An urban emphasis brings out the local complexities of exposure to heat while also considering the city’s entire administrative area. Finally, the climate justice literature on India has focused on adaptive planning for heat stress [43], and the need to include local constituencies in central government-led international negotiations on greenhouse gas emissions [44,45,46]. Our climate justice analysis of urban heat and greenspace in Delhi extends these studies by recognizing social vulnerabilities that become significant to building inclusive climate change policies and politics.

## 2. Materials and Methods

### 2.1. Study Area

**Figure 1:**
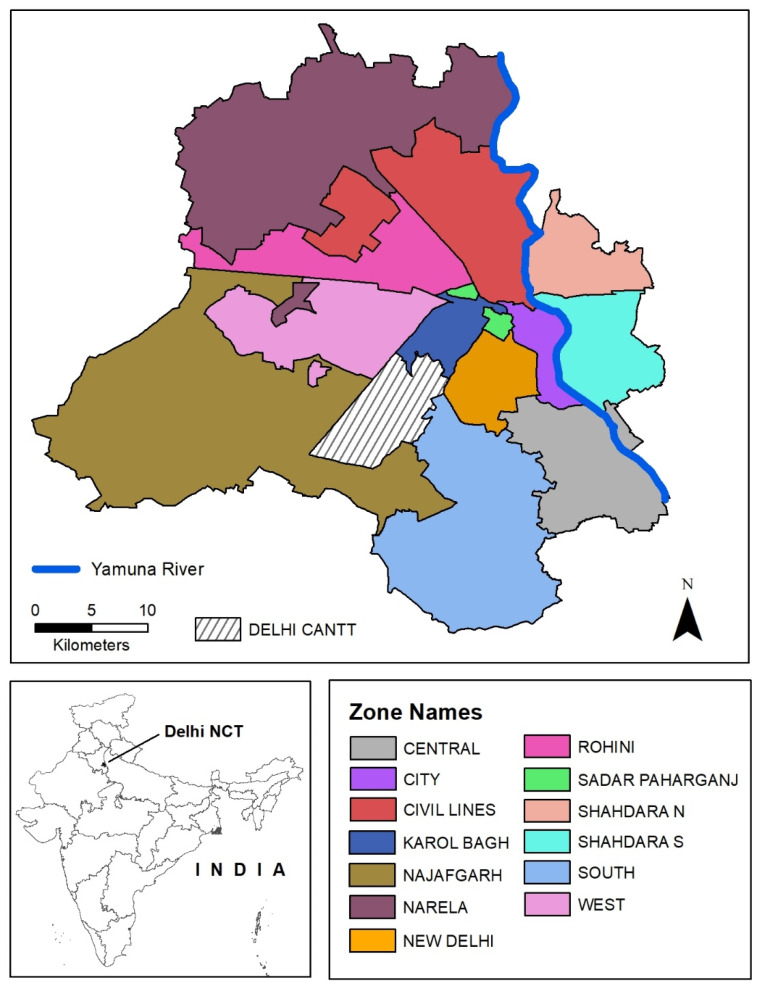
Location of study area (National Capital Territory of Delhi) and zones, 2011.

The National Capital Territory (NCT) of Delhi is divided for administrative and electoral purposes into wards, which are the smallest spatial units for which socio-demographic data can be obtained from the latest Census (2011) for India’s cities. There were 281 wards in Delhi based on census data which the city’s two municipal governments had grouped into 13 zones (Figure 1). Thus, 272 wards under the Delhi Municipal Corporation (DMC) were classified into 12 zones, and the 9 wards of the New Delhi Municipal Corporation (NDMC) were grouped as the New Delhi zone. The Cantonment (Delhi Cantt) is a separate administrative unit under military jurisdiction and was not utilized for our statistical analysis due to lack of Census data.

The 2011 Census of India counted 16.8 million inhabitants in the NCT of Delhi, which is estimated to have risen to at least 23 million in 2021 [47]. Demographic projections of Delhi indicate that it will rank as the largest urban agglomeration in the world by 2030 [47,48]. In 2011, Delhi had the highest population density of any state or union territory of India at 11,320 persons per square kilometer, with the national average being 382 [49]. In terms of age, the 2011 population pyramid for the NCT of Delhi indicated 27% of the population is 14 years old or under, a little lower than around 30% for India as a whole [49,50]. Of the NCT’s population, 98% were classified as urban, while 78% of its land area was classified as urban [49]. The relatively rural parts of Delhi are the southwest and northwest districts that correspond to Najafgarh and Narela zones, respectively (Figure 1). In 2011, 94% of these two districts was classified as urban [49]. In terms of housing characteristics, the majority of the population in 2000 lived in unauthorized or regularized slums or colonies (65%), a small proportion lived in rural and urban villages (12%), and 24% lived in planned colonies with assured access to infrastructural services [51,52]. The combination of a rapidly expanding population, higher percentage of children, and presence of informal settlements (slums and colonies) highlights the need to understand social vulnerability to urban heat in Delhi.

### 2.2. Dependent Variables: UHRI and NDVI

We estimated separate dependent variables to assess urban heat exposure and greenspace coverage at the ward level in NCT of Delhi. Since both heat and greenspace were measured for May and September, this provided four dependent variables. Physical data relating to land surface temperature, surface imperviousness, and vegetation abundance were gathered from LANDSAT 5 satellite Thematic Mapper (TM) remotely sensed imagery taken 8 May and 29 September 2011. These data values have seven bands, with a spatial resolution of 30 m in the visible and short-wave infrared and near infrared bands, and 120 m in the thermal band. Clear sky imagery from these dates were selected for a pre-monsoon and late-monsoon seasonal contrast of the hottest month of May (high urban heat, low vegetation) with the cooler month of September (low urban heat, high vegetation), respectively. Due to heterogeneous land uses within a city as large as Delhi, the distribution of heat and vegetation may vary in different seasons. Evaluating changes of the physical data within the study area allows for a sensitivity analysis of the relationship between heat exposure, vegetation proximity, and social indicators. All values for urban heat and greenspace were calculated at the pixel level. The mean values from all pixels located within each ward boundary in the NCT of Delhi were utilized for our study.

Heat exposure was estimated using the urban heat risk index (UHRI), a composite index of biophysical factors related to urban heat. The UHRI was calculated using the equation: *UHRI = [LST (z score) + NDBI (z score)] − NDVI (z score)* [53]. LST (land surface temperature) was calculated from thermal data (band 6) utilizing the mono-window algorithm that is based on the thermal transference equation [54,55]. NDBI (normalized difference built-up index) measured impervious surface coverage, while vegetation abundance, indicative of greenspace, was assessed by calculating the NDVI (normalized difference vegetation index). LST as well as landscape factors of impervious surface coverage and vegetation abundance are strongly correlated with the urban heat island [56,57]. The UHRI thus takes all three into account as an indication of the spatial extent and intensity of the urban heat island [53].

Additionally, we utilized the NDVI component of the UHRI as a separate measure of vegetation abundance in May and September to isolate the role of greenspace in heat mitigation. The NDVI is a robust indicator that is readily calculable from multispectral imagery [58,59]. It has been used in prior environmental exposure studies as a proxy for the distribution of urban greenspace [60,61], and as an indicator of vegetation abundance in the analysis of green biomass and urban greenspace [62,63]. A separate analysis of NDVI became useful in the case of Delhi due to the specific geography of greenspace here, consisting of forested and riverine areas in the central and eastern parts, and agricultural land uses in the urban peripheries to the north and west.

### 2.3. Independent Variables

The independent variables utilized in our study represented three categories of social vulnerability: (i) socio-demographic vulnerability, denoted by children, caste, and family size; (ii) housing-related vulnerability, denoted by household access to assets, electricity, and home ownership; and (iii) employment-related vulnerability, denoted by literacy and involvement in agriculture. All of these independent variables are variables of interest since they measure unique aspects of social vulnerability. In addition, population density was used as a control variable. Since our study was mainly exploratory, it was expected that statistical analysis would reveal the significance of these variables to understanding social vulnerability with respect to the spatial distribution of heat and greenspace in Delhi.

Five of our independent variables were derived from the 2011 Primary Census Enumeration data. This included population density, or the total number of people per square km in each ward, which was used as a control variable in our multivariable models. Previous studies on the social distribution of urban heat have found population density to be positively associated with the UHRI in U.S. urban areas and also used it as a control variable [3,53]. The proportion of individuals aged 6 years or less was included to examine the relationship between urban heat and presence of young children. Children were considered to be a relatively powerless group in home location decisions and more susceptible to heat illness than adults for multiple reasons, including their greater surface area to body mass ratio, lower rate of sweating, and slower rate of acclimatization [64]. The proportion of the ward population classified as Scheduled Caste (SC) was used as a measure of social marginalization, since this classification refers to caste groups within Hindu, Buddhist, and Sikh religions who have faced social discrimination due to their lower status and associated occupational roles. This variable has been utilized to denote social marginalization in environmental justice studies in India [30,31,32]. We also used literacy rate, defined as the proportion of the population aged 7 years or more that was literate, to represent ward socioeconomic status. Since the Census of India does not provide data on annual income, poverty, or wages, literacy rate can be used to denote employability and hence as proxy for socioeconomic status [30]. The fifth variable included the proportion of workers in the ward who were involved in agricultural activities. This was estimated as the sum of main and marginal cultivators and agricultural laborers, divided by the total number of workers in the ward. Wards with higher values of this variable can be expected to have more residents involved in outdoor agricultural labor and hence be more vulnerable to the adverse effects of heat exposure [21,22].

We utilized four additional variables from the 2011 Houselisting and Housing Census. These included the proportion of households having availability of assets (television, computer/laptop, telephone/mobile phone, or scooter/car), those with electricity as the main lighting source, those living in a house they own, and those with household size of nine persons or higher. The availability of specified assets and electricity in the household can be expected to reflect both higher economic status and ability to mitigate heat exposure [32]. Although home ownership has been linked to greater wealth or assets in U.S. urban areas, its interpretation in India has to be nuanced to accommodate the contextual specificities of housing markets [31]. Owning houses in the NCT of Delhi is potentially associated with households involved in agricultural occupations which can include low and middle income households. New migrants with well-paying jobs can reside in rental housing units in economically affluent wards with lower heat exposure and greater proximity to greenspace. Finally, households with nine or more persons potentially reflected higher levels of crowding and lower economic affluence. However, the possible presence of higher income households residing in large family owned compounds could influence the relationship between larger household sizes and lower socioeconomic status.

### 2.4. Statistical Analysis

Our analysis encompassed 281 wards in the NCT of Delhi for which complete data on the aforementioned independent variables from the 2011 Census of India were available. The Delhi Cantonment was the only ward excluded due to data unavailability. We used a multivariable approach to analyze each of our dependent variables (May UHRI, September UHRI, May NVDI, and September NDVI) as a function of all independent variables in a single model. Our multivariable models are based on generalized estimating equations (GEEs) with robust covariance estimates, which extend the generalized linear model [65] to accommodate clustered data [66]. GEEs are suitable for this study because they relax several assumptions of traditional regression models, impose no strict distributional assumptions such as normality for the variables analyzed, and consider variable clustering across units of analysis—in this case, wards [67,68,69,70]. For estimating a GEE, clusters of observations must be specified which assume that observations from within a cluster are statistically related, while observations from different clusters are independent. Our cluster definition was based on the zone within which each ward is located (Figure 1), based on the assumption of dependence of wards within a specific zone. This approach yielded 13 clusters of wards, with a range of 6 to 36 wards per cluster. GEEs also require an intracluster dependency correlation matrix to be specified [70]. After considering several correlation structure specifications, the ‘unstructured’ specification was chosen for GEEs using UHRI as the dependent variable, and the exchangeable specification was chosen for GEEs using NDVI as the dependent variable.

For selecting the best-fitting model, we estimated a series of GEEs by modifying the model specifications. We explored normal, gamma, and inverse Gaussian distributions with log and identity link functions (six different specifications). An identity link function assumes the dependent variable is directly predicted and not transformed, while a log link function estimates the natural logarithm of the dependent variable. We selected the normal distribution with log link function for GEEs using UHRI as the dependent variable, and the normal distribution with an identity link function for GEEs using NDVI as the dependent variable. All independent variables were standardized before inclusion in the GEEs and standardized coefficients are presented in the table summarizing these models. The statistical significance of each individual variable coefficient was determined using two-tailed *p*-values from the Wald chi-square test. Finally, the multicollinearity condition index was calculated for the combination of independent variables included in each GEE. None of the models yielded a condition index higher than 5.0, indicating that these GEEs were not affected by multicollinearity. All statistical analyses were conducted using IBM SPSS Statistics (version 26) software (IBM, Armonk, NY, USA).

## 3. Results

**Figure 2:**
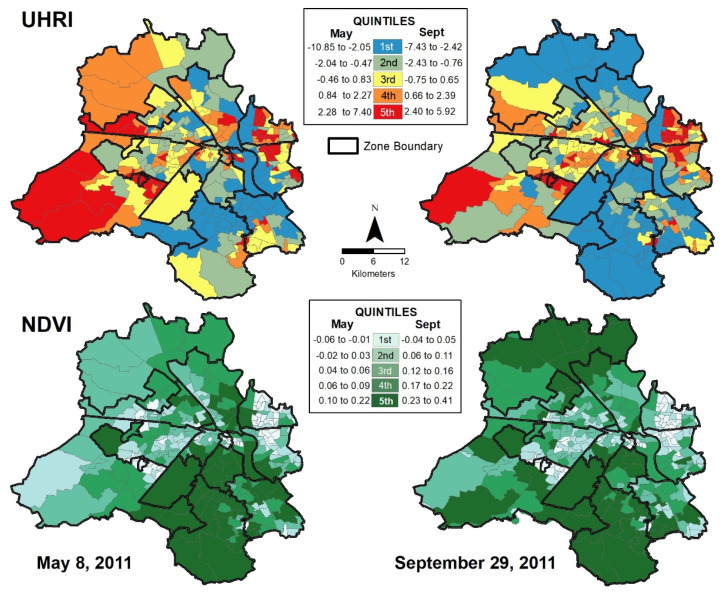
Ward level distribution of dependent variables for the NCT of Delhi. Top maps depict urban heat risk index and bottom maps depict NDVI, for 8 May and 29 September 2011 (Note: UHRI and NDVI calculated by author utilizing Landsat 5 TM imagery).

Before presenting the results of our statistical analysis, it is useful to consider the pre-monsoon and post-monsoon geographic distributions of the UHRI and NDVI in the NCT of Delhi. The ward level distributions of our dependent variables are shown as classified choropleth maps where wards are grouped into quintiles (Figure 2). Although UHRI values were higher in May, wards with greater UHRI in both May and September (highest quintile or top 20%) are located mainly in an east–west belt across the central part of the NCT of Delhi—including Najafgarh, West, Rohini, Narela, Civil Lines, and the Shahdara North and South zones. In contrast to these spatial patterns of the UHRI, wards with greater NDVI in both May and September are located in a north–south belt across the eastern part of the NCT, including the South, Central, New Delhi, and City zones. Southern Delhi shows especially high relative vegetation in both May and September, with the northern and southwestern peripheries of the NCT showing increases in September. The spatial distribution of lower UHRI and higher NDVI corresponds with the landscape factors of the Delhi Ridge and the Yamuna River. Higher vegetation patterns in September appear to spatially coincide with rural and agricultural areas in northern and southwestern Delhi.

Our statistical analysis comprises multivariable GEEs to model the relationship between each of the dependent variables and the set of independent variables described previously. Ward level descriptive statistics for all our dependent and independent variables are listed below (Table 1). While values of the UHRI vary considerably across the study area in both May and September, a wider range and higher variability can be observed in May. For values of the NDVI, the variability is greater in September than May—a potential reflection of post-monsoon vegetation increases in the northern and southwestern wards. With regard to the independent variables, the proportions of socially vulnerable groups such as children, SCs, and agricultural workers indicate relatively lower variability in their values. However, the proportions of households with assets and electricity, as well as those owning their house, indicate considerably higher variability and suggest substantial socioeconomic disparities across the NCT of Delhi.

Results from the GEE using May UHRI as the dependent variable includes beta coefficients and their 95% confidence intervals (CI), as well as the Wald chi-square statistic and its *p*-values (Table 2). Numbers in the Exp(Beta) column can be interpreted as the percentage change in the dependent variable (i.e., May UHRI) for every one standard deviation increase in each of the independent variables (after subtracting one and multiplying by 100). Controlling for the effects of clustering and other contextual factors, the May UHRI indicates a significant and positive association (*p* < 0.05) with the ward’s population density and the proportions of children, literate residents, agricultural workers, and households of larger size. More specifically, a one standard deviation increase in the proportion of children, literate residents, agricultural workers, and larger-size households is associated with approximately 67%, 151%, 64%, and 101% increases in values of the May UHRI, respectively. A significant and negative relationship with the dependent variable is observed in terms of proportions of households with assets and electricity (*p* < 0.01). A one standard deviation increase in the proportion of households with assets and those with electricity is associated with about 86% and 45% decreases in the May UHRI, respectively.

Results from the GEE using September UHRI as the dependent variable (Table 3) indicate a significantly positive association with the proportions of children and literate residents (*p* < 0.05), and a negative relationship with the proportions of SCs, households with assets, and households of larger size (*p* < 0.01). A one standard deviation increase in the proportion of children and literate residents is associated with approximately 54% and 91% increases in values of the September UHRI, respectively, while a one standard deviation increase in the proportion of SCs, households with assets, and households of larger size is associated with about 58%, 66%, and 45% decreases in the September UHRI, respectively. Although the proportions of agricultural workers and households with electricity revealed significantly positive and negative coefficients (*p* < 0.05), respectively, in the GEE for the May UHRI, both these variables yielded non-significant coefficients (*p* > 0.05) in the GEE for the September UHRI.

The GEE for the May NDVI (Table 4) indicates a significant and positive association with the proportion of SCs, households with assets, and households with electricity (*p* < 0.01). Since the GEEs for the NDVI are not based on a logarithmic function, numbers in the Beta column represent the percentage change in the dependent variable for every one standard deviation increase in each of the independent variables (after multiplying by 100). Specifically, a one standard deviation increase in the proportion of SCs, households with assets, and households with electricity is associated with 0.5%, 1.3%, and 0.8% increases in the May NDVI, respectively. A significantly negative relationship with this dependent variable (*p* < 0.05) is indicated by the literate proportion, home ownership, and larger-sized households. A one standard deviation increase in the proportion of literate residents, households owning homes, and households of larger size is associated with 1.1%, 0.5%, and 0.8% decreases in the May NDVI, respectively.

Results from the GEE using September NDVI as the dependent variable (Table 5) indicate a significantly positive association (*p* < 0.01) with the proportions of children, SCs, agricultural workers, households with assets, and households with electricity, but a negative relationship (*p* < 0.005) with the proportions of literate residents, households owning homes, and households of larger size. A one standard deviation increase in the proportion of children, SCs, agricultural workers, households with assets, and households with electricity is associated with about 1.7%, 0.9%, 1.0%, 2.4%, and 1.4% increases in values of the September UHRI, respectively, while a one standard deviation increase in the proportion of literate residents, households owning homes, and households of larger size is associated with about 2.0%, 0.9%, and 1.6% decreases in the September UHRI, respectively. Although the proportions of children and agricultural workers indicated a non-significant association with the NDVI in May (*p* > 0.30), both these independent variables show a significantly positive relationship (*p* < 0.01) with the September NDVI.

## 4. Discussion

This study extends research on climate justice by analyzing social inequities in the spatial distributions of: (1) urban heat exposure, based on the UHRI--an index of physical factors correlated with the urban heat island effect; and (2) proximity to urban greenspace based on the NDVI in the NCT of Delhi. In spatial terms, Delhi has a heterogeneous distribution of the UHRI and NDVI reflecting its complex urban morphology. At its eastern margin, Delhi contains agricultural flood plains and parkland along the Yamuna River characterized by lower heat exposure and higher levels of vegetation. Economically and socially marginalized groups of people are likely to live close to the river due to caste-based occupations [71]. Parallel to the Yamuna and to its west is the Delhi Ridge, which comprises a series of discontinuous outcrops of the Aravalli Hills. The afforestation of the Ridge was selectively undertaken to benefit governmental elites during British colonial rule, and even as it has been partially deforested since the 1950s, it remains a considerable greenspace within Delhi available as residential and leisure space for affluent residents [72,73]. Delhi’s agricultural areas in its north and west also become useful in understanding heterogenous exposure to heat and greenspace. In pre-monsoon May, these areas are in the highest quintile of heat exposure, and the western-most wards remain high in September compared to the rest of Delhi. However, in the post-monsoon period, these northern and western areas show vegetation levels that match or exceed the Yamuna and Ridge areas, possibly due to crop growth. Not only do variations in pre-monsoon and post-monsoon greenspace result in differential heat exposure, these have also sorted the residential population into neighborhoods based on socioeconomic status, the key factor influencing social vulnerability.

With respect to the UHRI in May, the hottest month of the year, we found several vulnerable groups such as children, agricultural workers, and larger sized households to be significantly overrepresented in wards with greater heat exposure, after controlling for spatial clustering, population density, and other relevant factors. With regard to social vulnerability related to economic status, our multivariable analysis revealed that households with assets and electricity are significantly underrepresented in neighborhoods with greater heat exposure in May. This suggests that households with fewer resources or reduced capacity to mitigate heat-related risks (e.g., via home air-conditioning) face disproportionately higher heat exposure. A study in the U.S. also found that socioeconomically disadvantaged residents faced significantly greater exposure to urban heat [9]. The prevalence of agricultural workers in wards with higher pre-monsoon UHRI is especially noteworthy, since employment in outdoor labor is a key risk factor in heat exposure [21,22]. Given that the proportion of agricultural workers was significantly and positively related to UHRI in May, the need to consider heat stress experienced by agricultural workers becomes important in the case of Delhi.

Although temperatures and UHRI values decline considerably after the monsoon, wards with a higher proportion of children and lower proportion of households with assets were still found to face significantly greater heat exposure in September. However, proportion of agricultural workers revealed a non-significant association with September UHRI, which suggests a potential reduction of the adverse heat-related impacts in comparison to May.

With regard to pre-monsoon vegetation, vulnerable groups such as children, agricultural workers, and larger size households that face significantly higher heat exposure were found to reside in wards with a non-significant or negative relationship with the May NDVI. Economically affluent groups such as households with assets and those with electricity that indicated significantly lower heat exposure in May were found to reside in wards with significantly higher greenspace based on the NDVI. These results are consistent with a previous U.S. study in which socioeconomically advantaged groups had greater access to urban greenspaces [10]. It is noteworthy that SCs, considered to be a socially vulnerable group, are linked to higher greenspace which could be related to their concentration in areas proximate to the Yamuna river. Households owning homes were significantly associated with lower greenspace in May, and this finding potentially reflects the absence of lower or moderate income residents in wards characterized by expensive rental housing and higher greenspace.

Our multivariable analysis of the NDVI in September yielded results that were similar to those observed in May, with two exceptions. The proportions of children and agricultural workers indicated a non-significant association with the NDVI in May, but both these variables showed a significantly positive relationship with the NDVI in September. For these two vulnerable groups, our findings thus suggest higher protection from heat exposure via greenspace in September, but no significant protection from heat exposure in the hotter or pre-monsoon month of May.

Overall, our statistical findings provide substantial evidence to suggest that exposure to heat in Delhi is shaped by demographic vulnerability in terms of the proportions of children and households with 9 or more members, economic vulnerability in terms of lack of access to assets and electricity, and employment vulnerability related to agricultural work. Greenspace as measured by the NDVI is also related to economic factors, specifically households with assets and electricity. These findings collectively suggest that climate change vulnerability in Delhi will be defined by a combination of presence of children, household socioeconomic status, as well as dependence on agricultural occupations. In terms of the latter, Delhi’s constant growth into surrounding rural areas, and strong agricultural traditions due to location in India’s northern Green Revolution belt, shapes vulnerability to heat in terms of both economic characteristics and social identities.

In policy terms, our study shows that climate change as evidenced by rising levels of urban heat will disproportionately impact households that lack assets and electricity, so that socioeconomic status remains useful in terms of identifying those who need assistance to mitigate heat stress. Additionally, there is a need to expand electricity service to underserved populations to ensure that they have some access to cooling appliances. Another important group to focus on are young children, so that policies to make cooling facilities available in local schools might also be considered. Agricultural laborers are also vulnerable to heat in the pre-monsoon period, and this points to the need to pay attention to populations on the urban periphery instead of focusing only on the central city. More broadly, there is an urgent need to expand greenspace and access to electricity to ensure that heat mitigation options become available to all.

There are several limitations to this study that are important to consider, some of which can be addressed in future research. First, the NDVI can be a poor indicator of biomass when there is little groundcover, such as in the semi-arid areas to the west of Delhi, and lose sensitivity when there is dense leaf cover [74,75]. This problem can be circumvented by applying image classification and high-resolution aerial imagery [76]. However, it should be noted that our study did not completely warrant the application of image classification methods due to the desirability of capturing an image comparable to other data used in calculating UHRI. Second, our analysis was based on ward level data and socio-demographic variables available in the Census of India. The use of additional local information and household-level surveys could help clarify factors influencing statistical relationships reported in this article, and determine the ways in which households residing in specific neighborhoods of Delhi were negatively impacted by heat exposure and greenspace absence. Third, it should be noted that our statistical findings are based on environmental and socio-demographic variables from 2011 which represents the latest year for which population data are currently available in the Census of India. We therefore used remotely sensed imagery from the same year to avoid a temporal mismatch. While more recent data on urban heat and vegetation abundance might indicate higher heat risk and lower greenspace in specific areas, the overall spatial distribution patterns of these indicators are unlikely to have changed significantly over time [16]. Finally, since our study focuses on a single urban area (i.e., NCT of Delhi) located within India’s northern agricultural belt, our findings may not be applicable to other cities located in other regions of India. However, the simultaneous consideration of heat exposure, greenspace presence, and social vulnerability factors provides a useful conceptual and analytical framework for examining climate justice within and beyond urban India.

## 5. Conclusions

This article has documented how the spatial distribution of heat and greenspace relates to the spatial distribution of social vulnerability at the ward level in Delhi. Our statistical results reveal that several variables denoting social vulnerability are significantly (*p* < 0.05) related to both UHRI and NDVI. Specifically, our multivariable models indicate that the May UHRI has a positive relationship with proportions of children and agricultural workers, and a negative relationship with proportions of households with assets and those with electricity. In contrast, the May NDVI is not significantly associated with the proportions of children and agricultural workers, while it shows a positive relationship with proportions of households with assets and electricity. In September, the proportion of agricultural workers shows an interesting shift to a positive relationship with NDVI possibly due to increase in planted crops. These findings from Delhi collectively reveal that vulnerable demographic and economic groups are less likely to be able to mitigate heat stress as they comprise young children, outdoor agricultural workers, and households lacking assets and electricity, so that a situation of climate injustice is very clearly demonstrated. Poised to become the most populous city in the world by 2030 [47], it is likely that impervious surfaces in Delhi will continue to expand leading to further intensification of the urban heat island effect and loss of greenspace. Our study therefore makes a valuable contribution to incorporating climate justice considerations in Delhi’s climate change planning by demonstrating which vulnerable groups should be prioritized in policies related to mitigation of heat stress.

## Figures and Tables

**Table 1:** Summary statistics for variables analyzed (*n* = 281 wards).

	Min	Max	Mean	SD
**Dependent variables:**				
May urban heat risk index (UHRI)	−10.910	7.498	0.001	2.682
Sept UHRI	−5.163	4.657	−0.001	1.834
May normalized difference vegetation index (NDVI)	−0.059	0.199	0.043	0.053
Sept NDVI	−0.037	0.408	0.142	0.099
**Independent variables:**				
Population density (persons per sq. km)	179	184,468	27,840	23,414
Proportion children (age 6 years or less)	0.058	0.160	0.116	0.021
Prop Scheduled Caste	0.002	0.720	0.169	0.115
Prop literate (age more than 6 years)	0.720	0.971	0.866	0.055
Prop workers involved in agriculture	0.001	0.130	0.010	0.016
Prop households (HHs) with specified assets *	0.001	0.725	0.236	0.176
Prop HHs with electricity as lighting source	0.283	1.000	0.947	0.151
Prop HHs owning their house	0.000	0.906	0.636	0.182
Prop HHs of size 9 persons and above	0.016	0.153	0.056	0.024

* Includes television, computer/laptop, telephone/mobile phone, and/or car/scooter.

**Table 2:** Generalized estimating equation for predicting May UHRI using ward level socio-demographic variables.

	Beta (*p*-Value)	Lower 95% CI	Upper 95% CI	Exp (Beta)	Wald Chi-Sq.
Population density	0.516 (0.002) **	0.187	0.846	1.675	9.417
Proportion children	0.922 (0.024) *	0.120	1.724	2.514	5.074
Prop Scheduled Caste	−0.110 (0.406)	−0.370	0.150	0.896	0.690
Prop literate	0.495 (0.001) **	0.202	0.788	1.640	10.965
Prop workers in agriculture	0.394 (0.016) *	0.074	0.714	1.483	5.815
Prop HHs with specified assets	−1.978 (0.017) *	−3.596	−0.359	0.138	5.737
Prop HHs with electricity	−0.605 (0.010) *	−1.068	−0.143	0.546	6.577
Prop HHs owning their house	0.133 (0.778)	−0.790	1.055	1.142	0.070
Prop HHs of size 9 and above	0.696 (0.017) *	0.124	1.269	2.006	5.685
Intercept	−2.112 (0.062)	−4.329	0.105	0.121	3.487
Scale	0.696				
Model fit (QIC)	1845.262				
N (wards)	281				

* *p* < 0.05, ** *p* < 0.01.

**Table 3:** Generalized estimating equation for predicting September UHRI using ward level socio-demographic variables.

	Beta (*p*-Value)	Lower 95% CI	Upper 95% CI	Exp (Beta)	Wald Chi-Sq.
Population density	1.182 (0.000) ***	0.813	1.551	3.261	39.352
Proportion children	0.434 (0.023) *	0.060	0.808	1.543	5.171
Prop Scheduled Caste	−0.862 (0.001) **	−1.042	−0.682	0.422	88.097
Prop literate	0.649 (0.003) **	0.224	1.073	1.914	8.976
Prop workers in agriculture	−0.012 (0.937)	−0.321	0.296	0.988	0.006
Prop HHs with specified assets	−1.084 (0.000) ***	−1.310	−0.857	0.338	87.998
Prop HHs with electricity	0.307 (0.064)	−0.017	0.632	1.359	3.442
Prop HHs owning their house	0.033 (0.825)	−0.260	0.326	1.034	0.049
Prop HHs of size 9 and above	−0.596 (0.005) **	−1.013	−0.179	0.551	7.848
Intercept	0.536 (0.060)	−0.023	1.094	1.709	3.534
Scale	3.464				
Model fit (QIC)	1051.501				
N (wards)	281				

* *p* < 0.05, ** *p* < 0.01, *** *p* < 0.001.

**Table 4:** Generalized estimating equation for predicting May NDVI using ward level socio-demographic variables.

	Beta (*p*-Value)	Lower 95% CI	Upper 95% CI	Wald Chi-Sq.
Population density	−0.028 (0.000) ***	−0.037	−0.019	39.134
Proportion children	0.001 (0.633)	−0.004	0.007	0.228
Prop Scheduled Caste	0.005 (0.000) **	0.002	0.008	9.125
Prop literate	0.011 (0.001) **	−0.017	−0.004	10.870
Prop workers in agriculture	0.002 (0.356)	−0.002	0.007	0.852
Prop HHs with specified assets	0.013 (0.010) **	0.003	0.023	6.650
Prop HHs with electricity	0.008 (0.006) **	0.002	0.013	7.670
Prop HHs owning their house	−0.005 (0.030) **	−0.010	0.000	4.710
Prop HHs of size 9 and above	−0.008(0.003) **	−0.013	−0.003	8.633
Intercept	0.043 (0.000) **	0.033	0.054	64.430
Scale	0.001			
Model fit (QIC)	32.637			
N (wards)	281			

** *p* < 0.01, *** *p* < 0.001.

**Table 5:** Generalized estimating equation for predicting September NDVI using ward level socio-demographic variables.

	Beta (*p*-Value)	Lower 95% CI	Upper 95% CI	Wald Chi-Sq.
Population density	−0.046 (0.000) ***	−0.062	−0.030	32.202
Proportion children	0.017 (0.008) **	0.004	0.029	7.127
Prop Scheduled Caste	0.009 (0.005) **	0.003	0.015	7.840
Prop literate	−0.020 (0.000) ***	−0.028	−0.011	21.364
Prop workers in agriculture	0.010 (0.000) ***	0.005	0.015	14.746
Prop HHs with specified assets	0.024 (0.000) ***	0.016	0.032	33.704
Prop HHs with electricity	0.014 (0.000) ***	0.006	0.023	12.223
Prop HHs owning their house	−0.009 (0.002) **	−0.014	−0.003	9.962
Prop HHs of size 9 and above	−0.016 (0.000) ***	−0.022	−0.010	24.834
Intercept	0.145 (0.000) ***	0.124	0.166	180.852
Scale	0.005			
Model fit (QIC)	1051.501			
N (wards)	281			

** *p* < 0.01, *** *p* < 0.001.

## Data Availability

The data presented in this study are available on request from the corresponding author.
